# Mapping of nursing interventions for elderly women with vulnerability related to HIV/AIDS

**DOI:** 10.1590/1980-220X-REEUSP-2021-0360

**Published:** 2022-02-09

**Authors:** Márcia Cristina de Figueiredo Santos, Greicy Kelly Gouveia Dias Bittencourt, Patrícia Josefa Fernandes Beserra, Maria Miriam Lima da Nóbrega

**Affiliations:** 1 Universidade Federal da Paraíba, Programa de Pós-Graduação em Enfermagem, João Pessoa, PB, Brazil.; 2 Universidade Federal da Paraíba, Programa de Mestrado Profissional em Gerontologia, João Pessoa, PB, Brazil.

**Keywords:** Nursing Care, Standardized Nursing Terminology, Vocabulary, Controlled, Health Information Interoperability, Women’s Health, HIV, Atención de Enfermería, Terminología Normalizada de Enfermería, Vocabulario Controlado, Interoperabilidad de la Información en Salud, Salud de la Mujer, VIH, Cuidados de Enfermagem, Terminologia Padronizada em Enfermagem, Vocabulário Controlado, Interoperabilidade da Informação em Saúde, Saúde da Mulher, HIV

## Abstract

**Objective:**

To map the nursing interventions of the Terminology Subset for elderly women with HIV/AIDS-related vulnerabilities in the International Classification for Nursing Practice 2019/2020, according to the guidelines of the ABNT Standard *ISO/TR* 12.300/2016.

**Method:**

This is a descriptive exploratory study of terminological mapping, in which interventions underwent the technique of validation by consensus and human mapping. Interventions reaching 100% agreement regarding practical usefulness and classification in the Theory of Nursing Systems were validated. Finally, human mapping was performed with a single purpose and oriented from source concepts to target concepts.

**Results:**

A total of 218 interventions were validated. Following mapping, the numbers were updated due to the cardinality relationship, resulting in 221 interventions, 170 of which are not, and 51 are included in the International Classification for Nursing Practice 2019/2020.

**Conclusion:**

Mapping of the Terminological Subset of the International Classification for Nursing Practice 2019/2020 culminated in the review and update of the proposed terminology, and confirmed the usefulness of the classification system through pre-coordinated concepts.

## RESUMO

**Objetivo:**

Mapear as intervenções de enfermagem do Subconjunto terminológico para mulheres idosas com vulnerabilidades relacionadas ao HIV/Aids junto à Classificação Internacional para a Prática de Enfermagem 2019/2020, segundo as diretrizes da Norma ABNT *ISO/TR* 12.300/2016.

**Método:**

Estudo exploratório descritivo, de mapeamento terminológico, no qual as intervenções passaram pela técnica de validação por consenso e mapeamento humano. Foram validadas as intervenções que atingiram 100% de concordância em relação à utilidade prática e à classificação na Teoria dos Sistemas de Enfermagem. Por último, executou-se o mapeamento humano com propósito único e direção dos conceitos-fonte aos conceitos-alvo.

**Resultados:**

Totalizaram-se 218 intervenções validadas. Após o mapeamento, os números sofreram atualização devido à relação de cardinalidade, resultando em 221 intervenções, sendo 170 não constantes e 51 constantes na Classificação Internacional para a Prática de Enfermagem 2019/2020.

**Conclusão:**

O mapeamento do Subconjunto Terminológico junto à Classificação Internacional para a Prática de Enfermagem 2019/2020 culminou com a revisão e atualização da terminologia proposta, além de ratificar a utilidade do sistema de classificação por meio dos conceitos pré-coordenados.

## RESUMEN

**Objetivo:**

Mapear el subconjunto terminológico de intervenciones de enfermería para mujeres ancianas con vulnerabilidades relacionadas con el VIH/SIDA según la Clasificación Internacional para la Práctica de Enfermería 2019/2020, de acuerdo con los lineamientos de la Norma ABNT ISO/TR 12.300/2016.

**Método:**

Estudio descriptivo exploratorio de mapeo terminológico, en el que las intervenciones pasaron por la técnica de validación por consenso y mapeo humano. Se validaron las intervenciones que alcanzaron un 100% de acuerdo en cuanto a utilidad práctica y clasificación en la Teoría de los Sistemas de Enfermería. Finalmente, el mapeo humano se realizó con un solo propósito y dirección desde los conceptos de origen hasta los conceptos de destino.

**Resultados:**

Un total de 218 intervenciones fueron validadas. Después del mapeo, los números fueron actualizados debido a la relación de cardinalidad, dando como resultado 221 intervenciones, de las cuales 170 no están y 51 están en la Clasificación Internacional para la Práctica de Enfermería 2019/2020.

**Conclusión:**

El mapeo del Subconjunto Terminológico con la Clasificación Internacional para la Práctica de Enfermería 2019/2020 culminó con la revisión y actualización de la terminología propuesta, además de constatar la utilidad del sistema de clasificación a través de conceptos precoordinados.

## INTRODUCTION

Recent epidemiological data express a reduction in the sex ratio among the population affected by HIV/AIDS, characterizing the process of feminization of the epidemic, associated with the progression of the number of elderly people affected by the infection, where the reduction in the sex ratio is even more expressive, with evidence shown in the last epidemiological bulletin published that, in 2019, the age group with the lowest ratio was that of 50 years or more, with a ratio (M:F) of 1.7^(1)^.

The lack of specificity in public health policies, added to the vulnerability of elderly women to HIV infection, show the relevance of planning nursing care in an up-to-dated, systematized, and targeted manner. Thus, aiming to develop a set of nursing diagnoses/outcomes (NDs/NOs) and interventions (NIs) for specialized care for elderly women with vulnerabilities to HIV/AIDS, a proposal was developed for a terminological subset of the ICNP^®^ based on the Self-care Nursing Theory (SCNT) by Dorothea Orem^(2)^ and in Ayres’ conceptual framework of vulnerability^(3)^, whose objective is to support the planning of care based on identifiable determinants and to favor the systematic record of nursing care for this specific clientele^(4)^, as recommended by the International Council of Nurses (IEC) regarding the development of classification systems^(5)^.

The aforementioned subset consists of the following items: Message to readers; Importance for Nursing; Insertion of Nursing in the theoretical models of the study, Orem’s Self-Care Nursing Theory, and the conceptual framework of vulnerability; NDs/NOs Statements^(4)^, and NI statements, based on a term database for nursing practice with elderly women with HIV/AIDS^(6)^.

SCNT is subdivided into three theoretical constructs: Self-Care Theory (SCT), Self-Care Deficit Theory (SCDT) and Nursing Systems Theory (NST). NST, a theoretical subsidy used to categorize nursing interventions in the aforementioned subset, consists of help and support methods developed by nurses and classified as a wholly compensatory system (WC), where self-care actions shall be developed by nurses; partially compensatory system (PC), where the nurse and the patient are responsible for carrying out self-care; and support-education system (SE), which refers to the execution of therapeutic self-care activities by the individual and/or caregiver, after receiving educational instructions from the nurse to do so^(2)^.

As for the conceptual framework of vulnerability^(3)^, in the individual modality, cognitive and behavioral issues are addressed; in the social, contextual aspects of access to information, possibilities of incorporating them to practical changes, and the coping with cultural and social barriers are involved; and in the programmatic approach, commitment from authorities, as well as organized policies and actions, involving the ways in which health services work to reduce vulnerability^(3,7)^.

Classification systems, which help in the description and communication of nursing practice, standardizing the language, undergo constant updates depending on natural scientific evolution, and mappings are the processes used to allow collection and reuse of data for different purposes, whether they are providing a basis for research or health planning^(8–9)^. Thus, the importance of supporting the documentation of specific nursing care through the updated registry is recognized, justifying the development of the mapping of nursing interventions along with the latest version of the ICNP^®^.

Among the known mapping techniques, human mapping specifically is convenient to support the crossing of source and target data. Therefore, knowledge and human skills are required to relate concepts of different terminological resources individually, consisting of the mapping modality, considered more efficient for the analysis of shared meanings, and being able to use electronic support resources^(8–9)^.

In order to operationalize the terminological subset, which was structured based on the ICNP^®^ 2015 version, and knowing that the performance of a new mapping is recommended, as a mechanism for identifying and tracking new versions of target concepts to support document updating^(8)^, the following objective emerged: To map the nursing interventions of the terminology subset for elderly women with HIV/AIDS-related vulnerabilities at the ICNP^®^ 2019/2020, according to ISO/TR 12.300/2016 guidelines.

## METHOD

### Design of Study

Descriptive exploratory study of terminological mapping.

### Population and Local

Nursing interventions underwent consensus validation, which recommends the formation of a group consisting of the investigator nurse, considered the leader, and three to five clinical experts^(10)^. Therefore, a group of four investigators/nurses participating in the study was formed, configuring an intentional, convenience, and non-probabilistic sampling, recruited through an invitation letter via e-mail to 5 investigators/nurses about the stages of the study and volunteers, to which only 4 responded agreeing with the participation.

### Selection Criteria

Study participants were selected according to the following criteria: being a nurse, investigator, participating in a research group, having as minimum education a master or doctorate degree, and/or being a clinical nurse and/or being involved in teaching and /or research in the areas of HIV/AIDS and/or the elderly and/or ICNP^®^.

### Data Collection

The collection began with the availability of the validation instrument, in printed format, containing 261 interventions proposed in the terminological subset, as well as the Free Informed Consent Form (FICF), to the four experts who agreed to participate in the study for individual analysis, with 3 months prior to consensus. The validation process was continued and concluded with an in-person meeting held in January 2017, which was attended by everyone and lasted approximately one hour. These interventions were categorized based on the NST, within the WC, PC and SE systems.

Nursing interventions that reached a consensus of 100% agreement among specialists regarding practical usefulness and classification within nursing systems with marking of checkbox and persuasive discussion were considered validated. Specialists, to reach a consensus, could discuss when they disagreed on some aspect and, whenever adjustments were made in the composition of the interventions as requirements for their validation (alteration of terms of the action axis, of NI sequencing by order of priority of implementation and/or in the categorizations), they were performed.

Finally, human mapping was performed with a single purpose and orientation from the source concepts to the target concepts (historical terminological tracking of the subset’s nursing interventions in relation to ICNP^®^ pre-coordinated concepts). For this purpose, two specific worksheets were created in the Excel for Windows, one for the nursing interventions contained in the terminological subset and the other for the pre-coordinated concepts of ICNP^®^, mapping them by cardinality as an indicator of the degree of aggregation, showing the mapping relationships based on the demonstration of the level of equivalence, according to ISO 12.300/2016 guidelines, originating the list of interventions present in the ICNP^®^.

### Data Analysis and Treatment

The analysis of the mapping level of equivalence was guided by the assessment scale of meanings proposed by ISO 12.300, in which 1 means equivalence of meaning between concepts, besides lexical and conceptual equivalence; 2 means equivalence of meaning between concepts, but with synonymy; 3 means that the source concept is broader and has less specific meaning than the target concept/term; 4 means that the source concept is more restricted and has more specific meaning than the target concept/term; and 5 shows that no mapping is possible between the target and source concepts/terms, in which a concept with some level of equivalence was not found in the target^(8)^.

The subset NIs were replaced by the pre-coordinated concepts of the 2019/2020 ICNP^®^ that fall under equivalence relationships 1 and 2. The NIs classified as equivalence 3 and 4 were not replaced by the concepts of 2019/2020 ICNP^®^, with which they established a relationship, as they have a broader or more specific meaning, respectively, and thus do not have their characteristics accurately contemplated; therefore, with the NIs with cardinality relationship 5 in what regards to ICNP^®^ target terms/concepts, did not change and were kept as non-included NIs.

### Ethical Aspects

This study was approved by the Research Ethics Committee of the Health Sciences Center of Universidade Federal da Paraíba, under Opinion 853.001, in 2014, with recent approval of a new Opinion no. 4.429.145, in 2020, for continuity of the study. All ethical aspects related to research with human beings were respected, in accordance with Resolution nº 466/2012 of the National Health Council, with participants signing the consent form.

## RESULTS

A total of 218 interventions were validated among the 261 submitted for validation, representing approximately 83.5% of the outlined interventions, which made up the terminological subset. Among the validated interventions, 149 were classified as they meet the elderly’s health needs in the context of individual vulnerability to HIV/AIDS, of which 65 were directed to meet the nursing diagnoses of the health deviation requisite, 52 directed to meet the nursing diagnoses of the developmental requisite, and 32 directed to the nursing diagnoses of the universal requisite. Among these 149 NIs, 84 correspond to the SE system, 30 to the PC system and 35 to the WC system.

For the diagnoses validated in social vulnerability, 58 interventions (81.6%) were validated, 14 of which were aimed at meeting the diagnoses of the health deviation requisite, 8 of the developmental requisite, and 36 aimed at the diagnoses of the universal requisite. It should be noted that 27 of these interventions, designed to meet the self-care needs of social vulnerability, corresponded to the SE System, 19 to the WC System, and 12 to the PC Nursing Action System.

The total number of interventions validated in the programmatic context of vulnerability was 11 interventions (68.75%), 4 of which being aimed at meeting the diagnoses of the health deviation requisite and 7 of the universal requisite, 8 of which correspond to the WC System of nursing action and 3 of them to the SE System, with the PC System being excluded from this modality of vulnerability. The sequencing of validated interventions is also based on the specialits’ judgment as to the priority level of their implementation.

Prior to the mapping step, the NIs not included in the ICNP^®^ totaled 192 and the NIs listed in the ICNP^®^ a total of 14, of which 6 were repeated once (“Encourage the family’s involvement in the elderly’s health care”, “Stimulate adherence to the drug regimen”, “Inform the impact of the use of the drug on the patient’s lifestyle”, “Monitor symptoms and signs of infection”, “Perform Humor (or Laughter) Therapy” and “Use a calm and safe approach”) and two repeated 3 times each (“Request (or Require) feedback technique of the information provided ” and “Assess the client’s learning ability”), to meet the needs of different NDs.

**Chart 1:**
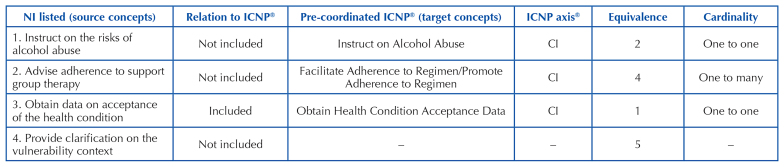
Cutout of human mapping of nursing interventions, with analysis of the level of equivalence between source concepts/terms and target concepts/terms – João Pessoa, Brazil, 2021.

After mapping the NIs in the 2019/2020 ICNP^®^, the numbers were updated, totaling 221 NIs (average of 4.25 for each ND), with 170 not included in the ICNP^®^ (sum of NIs with equivalence assessment 3, 4 and 5 in relation to the ICNP^®^ target terms/concepts) and 51 listed in ICNP^®^, the latter consisting of the sum of the 21 that fall in equivalence relation 1 to the 27 NIs that fall in the equivalence relation 2, plus three NIs that are fragmented into two each included in ICNP^®^, due to the cardinality of the human mapping of “one to many”^(8)^, as exemplified in Chart 1 below.

The result of the aforementioned NI equivalence analysis process included replacement of source statements for target statements, for example, in the **individual vulnerability**, the NI “Instruct on the risks of alcohol abuse” was replaced by the ICNP^®^ NI “Instruct on Alcohol Abuse”; the NI “Stimulate adherence to the drug regimen” was replaced by then NI “Promote Drug Adherence”; the NI “Control the environment to facilitate trust” was fragmented into the two interventions “Establish Trust” and “Environmental Therapy”, with which it established equivalence relation 2, among others. In **social vulnerability**, the changes were: the NI “Motivate family support” was replaced by the NI “Promote family support”; the NI “Establish a relationship of trust with the patient” was replaced by the NI “Establish Trust”; the NI “Perform Humor (or Laughter) Therapy” was replaced by the NI “Humor (or Laughter) Therapy”, among others. As for **programmatic vulnerability**, changes are summarized in the NI “Instruct on drug use” that was replaced by the NI “Instruct on medication” and the NI “Explain about the patient’s rights” which was replaced by the NI “Explain the Patient’s Rights”.

**Chart 2:**
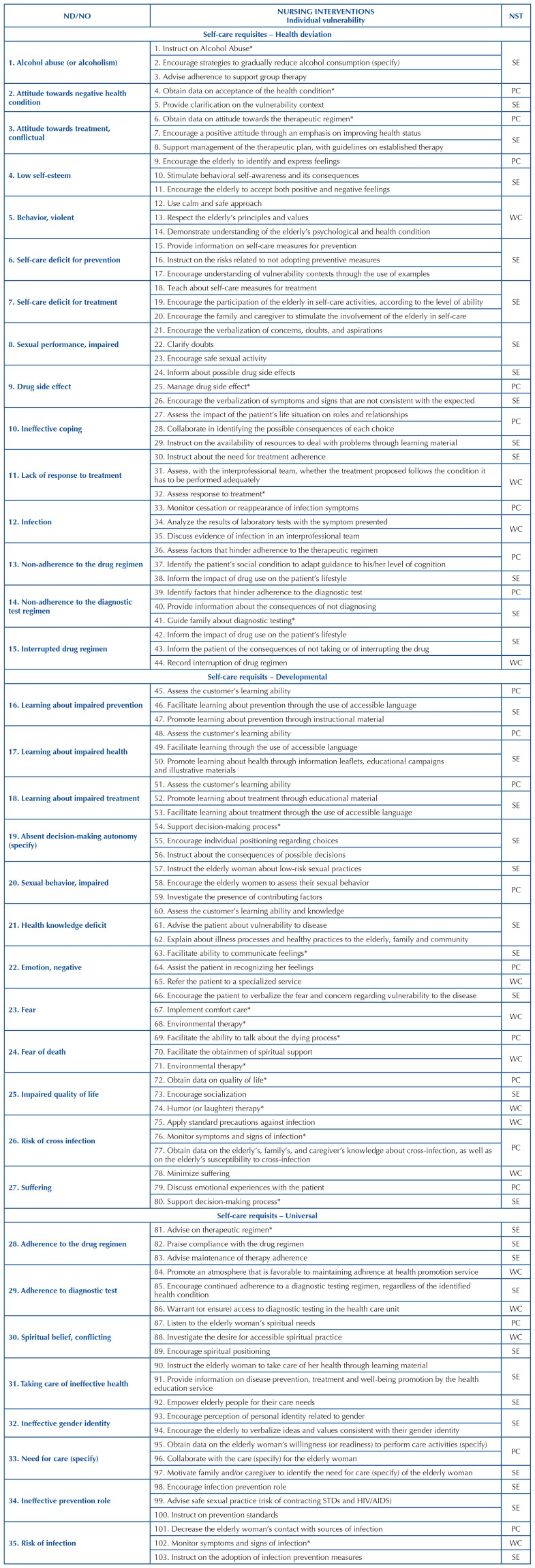
Cutout of the concepts of nursing interventions classified in the individual component of vulnerability and in the Nursing Systems Theory, in correspondence to the ND/NO of Orem’s self-care requisites – João Pessoa, Brazil, 2021.

Legend: *NIs listed in ICNP^®^.

**Chart 3:**
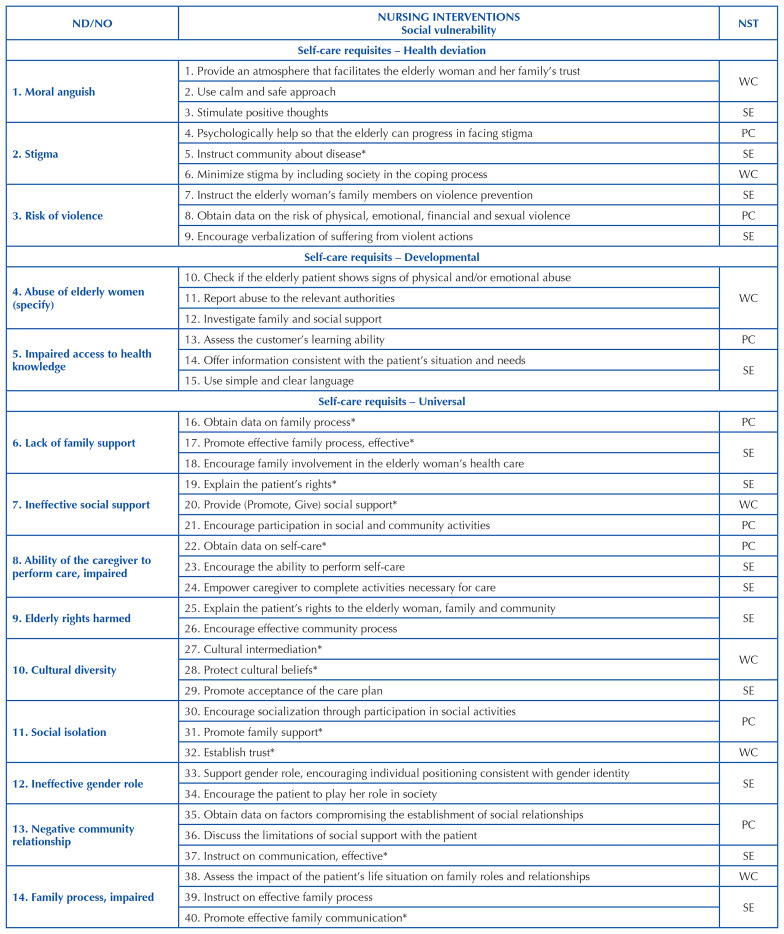
Cutout of the concepts of nursing interventions classified in the social component of vulnerability and in the Nursing Systems Theory, in correspondence to the ND/NO of Orem’s self-care requisites – João Pessoa, Brazil, 2021.

Legend: *NIs listed in ICNP^®^.

**Chart 4:**
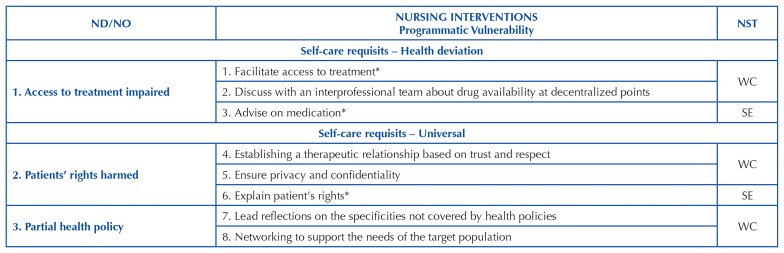
Cutout of the concepts of nursing interventions classified in the social programatic component of vulnerability and in the Nursing Systems Theory, in correspondence to the ND/NO of Orem’s self-care requisites – João Pessoa, Brazil, 2021.

Legend: *NIs listed in ICNP^®^.

Charts 2, 3 and 4 include cutouts of a maximum of 3 priority NIs for each ND, including some NIs that are included and others that are not included in the ICNP^®^ resulting from the mapping, as validated and categorized within the nursing systems.

## DISCUSSION

ICNP^®^ version 2019/2020 includes updates compared to the version that supported the structuring of the terminological subset, the 2015 version, among which a total of 105 new NI concepts, such as the NIs “Facilitate Learning” and “Promote Ability to Socialize”, as well as editorial change of four NI concepts^(11)^. Such changes demonstrate the importance of constantly updating terminologies that aim to standardize the practical professional language, so that they do not become obsolete, becoming opportunities to rescue innovative information^(12)^.

The aforementioned comparative rescue, favored by the mapping technique, is a didactic process for checking the relevance of professional decision-making arising from clinical reasoning^(13)^ and has been disseminated and used as an essential step in the structuring of ICNP terminological subsets^®^ (containing elements of nursing practice) in several areas of expertise, given the recognition of the need to adapt the terminologies under development to the revisions of the aforementioned classification^(12)^.

The highlight of this study’s mapping process is the *corpus* of 27 NIs initially not listed in the ICNP^®^ which fell into equivalence 2 in relation to the target concepts, as they portray a context of terms registered in different formats, but which have similar meanings, signaling the importance of conceptual uniformity/standardization that allows effective professional communication, as well as measurement and comparison of activities and results of the practice, contributing to the improvement of the care provided^(14)^ and consequent reduction in the vulnerability of elderly women to HIV/AIDS.

The emphasis that still falls on the three NIs that were fragmented into two NIs each, listed in the ICNP^®^, is related to the cardinality of “one-to-many”, as it consists of a principle derived from decision-making on the selection of one or more target-concepts representing a single source-concept^(9)^.

Reflecting the NIs in the light of the SCNT, they behave as resources that Nursing shall rely on to face the conditions of self-care deficits shown by the clientele through the NDs. As shown by the categorization of NIs, this coping can be initiated and completed by the nurse (WC), by the nurse in collaboration with the patient (PC), and also be performed by the patient after receiving adequate instructions for each care action (SE)^(2)^.

The rates of contamination of the elderly women by HIV/AIDS may be associated with sociocultural, programmatic, and/or individual factors, among which the influence of taboos and stereotypes on the sexuality of this group, the few opportunities in health services to discuss about sexuality with this clientele^(15)^ and about bodily changes in this age group^(16)^, the gender relations that limit decision-making for prevention^(15,17)^, the lack of health policies that meet the needs of that population^(18)^, as well as the lack of knowledge about the infection^(19)^, are perceived as factors that can increase the vulnerability of elderly women to HIV/AIDS and, in addition to being a phenomenon of interest to Nursing, are addressed in many of the NIs mapped and validated in this study for implementation by these professionals.

Although the number of NIs that aim to assist aspects of **individual vulnerability** in this group has been high in relation to other vulnerability modalities, in the categorization of interventions mapped among the concepts of nursing systems in the SCNT, this did not mean exclusive responsibility of the elderly woman for coping with and/or preventing HIV/AIDS infection. On the contrary, the quantitative data from the categorization of NIs (113 in the SE system and 63 in the WC system) showed the importance of the nurse’s role as a subject in the face of the elderly woman’s self-care demands.

The predominance of the classification of interventions in the **SE system** reflects the need for health actions aimed at providing information to the elderly, the family, and the caregiver. Whether in the social or individual sphere, the possibility of transforming the conditions that place elderly women in HIV/AIDS-related vulnerability is evident when conducting instructive actions to promote health and prevent diseases and injuries. Interventions aimed at this purpose are those based on encouragement, stimulus, guidance and health promotion^(20–21)^.

Regarding the **WC system**, there is a complexity of multidisciplinary health actions required by HIV/AIDS, which reflects the relevance of the forms of care developed by the Specialized Care Services (*SAE*). The multidisciplinary nature of the actions developed in these services includes the nurse as an important actor in the comprehensive care of the Person Living with HIV/AIDS (PLWHA) and consists of a means of support for the elderly person at all times of living with the virus^(22)^.

The nursing interventions proposed in this study, in addition to seeking to meet the needs of useful diagnoses for elderly women vulnerable to virus acquisition, aim to guide nursing care aimed at elderly women who are already living with HIV/AIDS, to emancipate them from the conditions of individual, social, and programmatic vulnerabilities to which they are exposed even when living with the virus, as well as to foster subsidies so that the continuity of nursing and multidisciplinary care becomes effective.

As for **social vulnerability**, the importance of recognizing organized civil society as capable of influencing the construction and implementation of public policies to fight the HIV/AIDS epidemic is observed. The social effects of epidemics can be mitigated or faced through the rupture of cultural and programmatic barriers that is allowed through the access of PLWHA to health services in general^(23)^.

Health education has an emancipatory potential against social vulnerability, so that, when knowing about forms of infection, prevention behaviors, diagnosis and treatment methods involving HIV/AIDS, there are great chances of transforming the conditions of vulnerability^(24)^. Discussions between professionals and older people on the topic of sexuality should be among routine health care actions^(21)^.

It is noticed that, if the instructive relationships allowed by the clarifying dialogue between health professionals and elderly women are distant, where a bond based on trust is not effectively established, it will be difficult to achieve good adherence to therapeutic regimens or diagnostic plans and follow-up, leading to impaired quality of life and a sequence of other self-care deficits.^(22)^.

In the context of **programmatic vulnerability**, not coincidentally, the highest frequency of interventions is found in the **WC system**, where it is restricted to the nurse/multiprofessional health team to act in a given situation so that it directs itself to effective solutions^(2)^. Considering panoramas of understanding the vulnerable context of some populations, in addition to individual accountability in prevention, coping and treatment, it is appropriate to approach social and institutional determinants, such as access to services and the professional look at sociocultural aspects as emancipatory mechanisms towards epidemics^(23)^. In this context, the theoretical categorization allows us to perceive the impossibility of outsourcing responsibility for the NIs, which should be assumed as the role of the nurse in the face of the demands of programmatic vulnerability, either through the SE system or through the WC system.

In spite of the scarcity of similar scientific literature that would allow to delineate the developed process, configuring itself as a limitation of the study, the mapping allowed the proposition of NIs considered useful for specialized nursing care.

## CONCLUSIONS

Human mapping of validated interventions from the ICNP^®^ Terminology Subset for elderly women with HIV/AIDS-related vulnerability, along with the pre-coordinated concepts of the 2019/2020 ICNP^®^, culminated in the revision and updating of the proposed terminology, allowing the establishment of relations ratifying the usefulness of ICNP^®^ through its pre-coordinated concepts, as well as the identification of the clientele’s specificities standing out from the referred classification system, but that represent care needs for prevention, promotion and health recovery. It is emphasized that the mapped NIs do not aim and should not limit the nurse’s therapeutic clinical reasoning, but only support the practice based on systematized care.

Such NIs should be subjected to operationalization, aiming at their clinical validation with the clientele of interest, so that it favors the development of terminology and the provision of specialized care, as well as stimulating nurses’ vision and practice to transformation of the contexts of vulnerability of this population.

## Financial support

This work was carried out with the support of the Coordenação de Aperfeiçoamento de Pessoal de Nível Superior – Brazil (CAPES) – Financing Code – 001.

## References

[1] Brasil. Ministério da Saúde. (2020). Secretaria de Vigilância em Saúde. Boletim epidemiológico de HIV e
Aids.

[2] Orem DE (2001). Nursing: Concepts of practice..

[3] Ayres JRCM, Czeresnia D, Freitas CM (2009). Promoção da saúde: conceitos, reflexões, tendências.

[4] Santos MCF, Nóbrega MML, Silva AO, Bittencourt GKGD (2018). Nursing diagnoses for elderly women vulnerable to
HIV/Aids. Rev Bras Enferm..

[5] Nóbrega MML, Cubas MR, Egry EY, Nogueira LGF, Carvalho CMG, Albuquerque LM, Cubas MR, Nóbrega MML (2015). Atenção primária em saúde: diagnósticos, resultados e intervenções de
enfermagem.

[6] Siqueira MCF, Bittencourt GKGD, Nóbrega MML, Nogueira JA, Silva AO (2015). Term base for nursing practices with elderly women with
HIV/Aids. Rev Gaucha Enferm..

[7] Malagón-Oviedo RA, Czeresnia D (2015). The concept of vulnerability and its biosocial
nature. Interface – Comunicação, Saúde, Educação..

[8] Associação Brasileira de Normas Técnicas. ISO/TR 12.300 (2016). Informática em saúde – princípios de mapeamento entre sistemas
terminológicos.

[9] Torres FBG, Gomes DC, Ronnau L, Moro CMC, Cubas MR (2020). ISO/TR 12300:2016 for clinical cross-terminology mapping:
contribution to nursing. Rev Esc Enferm USP..

[10] Kautz DD, Kuiper R, Pesut DJ, Williams RL (2006). Using NANDA, NIC, and NOC (NNN) language for clinical reasoning
with the Outcome-Present State-Test (OPT) model. Int J Nurs Terminol Classif..

[11] Garcia TR (2019). Classificação Internacional para a Prática de Enfermagem.
CIPE^®^: Versão 2019-2020..

[12] Cubas MR, Pleis LE, Gomes DC, Costa ECR, Peluci APVD, Shmeil MAH (2017). Mapping and definition of terms used by nurses in a hospital
specialized in emergency and trauma care. Revista de Enfermagem Referência..

[13] Morais SCRV, Nóbrega MML, Carvalho EC (2018). Cross-mapping of results and Nursing Interventions: contribution
to the practice. Rev Bras Enferm..

[14] Souza DR, Andrade LT, Napoleão AA, Garcia TR, Chianca TC (2015). Terms of International Classification for Nursing Practice in
motor and physical rehabilitation. Rev Esc Enferm USP..

[15] Aguiar RB, Leal MCC, Marques APO, Torres KMS, Tavares MTDB (2020). Idosos vivendo com HIV – comportamento e conhecimento sobre
sexualidade: revisão integrativa. Cien Saude Colet..

[16] Vieira KLF, Coutinho MPL, Saraiva ERA (2016). A sexualidade na velhice: representações sociais de idosos
frequentadores de um grupo de convivência. Psicologia: Ciência e Profissão..

[17] Bezerra VP, Serra MAP, Cabral IPP, Moreira MASP, Almeida AS, Patrício ACFA (2015). Preventive practices in the elderly and vulnerability to
HIV. Rev Gaucha Enferm..

[18] Pires PV, Meyer DEE (2019). Noções de enfrentamento da feminização da aids em políticas
públicas. Revista Polis e Psique..

[19] Araldi LM, Pelzer MT, Gautério-Abreu DP, Saioron I, Santos SSC, Ilha S (2016). Elderly with Human Immunodeficiency Virus: infection, diagnosis
and living with the disease. REME.

[20] Carvalho CMG, Cubas MR, Nóbrega MML (2018). Diagnósticos, resultados e intervenções de enfermagem no cuidado
às pessoas com estomia de eliminação intestinal. Estima – Brazilian Journal of Enterostomal Therapy..

[21] Lima ICC, Fernandes SLR, Miranda GRN, Guerra HS, Loreto RGO (2020). Sexualidade na terceira idade e educação em saúde: um relato de
experiência. Revista de Saúde Pública do Paraná..

[22] Casséte JB, Silva LC, Felício EEAA, Soares LA, Morais RA, Prado TS (2016). HIV/AIDS among the elderly: stigmas in healthcare work and
training. Revista Brasileira de Geriatria e Gerontologia..

[23] Alexander KA (2020). Social determinants of HIV/AIDS and intimate partner violence:
interrogating the role of race, ethnicity, skin color. Rev Lat Am Enfermagem..

[24] Aguiar RB, Leal MCC, Marques APO (2020). Knowledge and attitudes about sexuality in the elderly with
HIV. Cien Saude Colet..

